# Postoperative complications following colectomy for ulcerative colitis: A validation study

**DOI:** 10.1186/1471-230X-12-39

**Published:** 2012-04-27

**Authors:** Christopher Ma, Marcelo Crespin, Marie-Claude Proulx, Shanika DeSilva, James Hubbard, Martin Prusinkiewicz, Geoffrey C Nguyen, Remo Panaccione, Subrata Ghosh, Robert P Myers, Hude Quan, Gilaad G Kaplan

**Affiliations:** 1 Inflammatory Bowel Disease Clinic, University of Calgary, Calgary, Alberta, Canada; 2 Departments of Medicine, University of Calgary, Calgary, Alberta, Canada; 3 Community Health Sciences, University of Calgary, Calgary, Alberta, Canada; 4 Mount Sinai Hospital, University of Toronto School of Medicine, Toronto, Ontario, Canada; 5 Teaching Research and Wellness Center, 3280 Hospital Drive NW, 6D17, Calgary, AB, T2N 4N1, Canada

## Abstract

**Background:**

Ulcerative colitis (UC) patients failing medical management require colectomy. This study compares risk estimates for predictors of postoperative complication derived from administrative data against that of chart review and evaluates the accuracy of administrative coding for this population.

**Methods:**

Hospital administrative databases were used to identify adults with UC undergoing colectomy from 1996–2007. Medical charts were reviewed and regression analyses comparing chart versus administrative data were performed to assess the effect of age, emergent operation, and Charlson comorbidities on the occurrence of postoperative complications. Sensitivity, specificity, and positive/negative predictive values of administrative coding for identifying the study population, Charlson comorbidities, and postoperative complications were assessed.

**Results:**

Compared to chart review, administrative data estimated a higher magnitude of effect for emergent admission (OR 2.52 [95% CI: 1.80–3.52] versus 1.49 [1.06–2.09]) and Charlson comorbidities (OR 2.91 [1.86–4.56] versus 1.50 [1.05–2.15]) as predictors of postoperative complications. Administrative data correctly identified UC and colectomy in 85.9% of cases. The administrative database was 37% sensitive in identifying patients with ≥ 1Charlson comorbidity. Restricting analysis to active comorbidities increased the sensitivity to 63%. The sensitivity of identifying patients with at least one postoperative complication was 68%; restricting analysis to more severe complications improved the sensitivity to 84%.

**Conclusions:**

Administrative data identified the same risk factors for postoperative complications as chart review, but overestimated the magnitude of risk. This discrepancy may be explained by coding inaccuracies that selectively identifying the most serious complications and comorbidities.

## Background

Approximately 10% of ulcerative colitis (UC) patients require a colectomy within 10 years of diagnosis [1]. Colectomy for UC is a technically demanding operation associated with morbidity and mortality [2,3]. Patients undergoing elective procedures have lower risk of postoperative mortality, ranging from 0.0% to 1.0% [4-7]. In contrast, mortality in those requiring emergent colectomy was as high as 6.9% [8-10]. Other factors that have been shown to influence postoperative outcomes include older age and comorbidities [11].

Previous studies reporting postoperative outcomes in UC patients have used medical chart review to obtain clinical information. However, these studies are rarely population-based allowing for referral bias and have small sample sizes. Consequently, investigators have relied on administrative databases to study population-based estimates of postoperative outcomes [11]. Administrative databases are time and cost efficient resources but interpretation of results derived from administrative data is dependent on the validity of administrative coding for predictors and outcomes. Therefore, validation of these databases is a priority in health services research [12].

Although numerous studies have used administrative data to study UC outcomes [11,13,14], few have validated the accuracy of administrative data in identifying UC patients who underwent a colectomy. Furthermore, the accuracy of administrative data in characterizing risk factors such as comorbidities is inconsistent. Under-reporting of comorbidities is high[15] and differentiating postoperative complications from pre-admission comorbidities can be challenging. Consequently, inherent misclassification may be present when administrative databases are used to identify preoperative risk factors of postoperative complications [16].

Thus, we compared estimates of the preoperative risk factors (age, emergent colectomy, and comorbidities) associated with postoperative complications in UC patients undergoing colectomy, derived from two data sources: chart review and administrative data. Subsequently, we evaluated the accuracy of administrative databases in defining: 1) the UC study population; 2) preoperative risk factors; and 3) postoperative complications.

## Methods

### Study Population

The Data Integration, Measurement and Reporting Hospital Discharge Abstract Database (DAD) captures all hospitalizations in the Calgary Health Zone of Alberta Health Services, Canada. The Calgary Health Zone is a population-based health authority under a public, single payer system, with an estimated population of 1.3 million in 2009[17]. The DAD database used the *International Classification of Disease, Ninth Revision, Clinical Modification* (ICD-9-CM) up to March 31, 2001; ICD-10-CA and the *Canadian Classification of Health Intervention* (CCI) coding have been used since April 1, 2002.

The DAD was searched to identify adult patients (≥18 years) admitted to hospital between January 1, 1996 and December 31, 2007 with a diagnosis of UC (ICD-9 556.X, ICD-10 K51.X). We then identified UC patients who had a code for colectomy (ICD-9-CM 45.7, 45.8 or CCI: 1.NM.87, 1.NM.89, 1.NM.91, 1.NQ.89, 1.NQ. 90). Recognizing that the administrative database may have missed some UC patients who underwent a colectomy, we identified a cohort of patients admitted for an UC flare without a colectomy. All patients with UC at the primary diagnosis coding field and a random subset of patients with UC coded in the second or third diagnostic position were identified. All medical charts of patients identified by the administrative database were reviewed using a standardized, *a priori* defined electronic data extraction form. Data was extracted by five trained research assistants who were blinded to the original administrative coding. Fifty patient charts were used as a reference standard; all reviewers extracted data on these fifty charts and their abstraction was verified by a gold-standard reference data abstracter (SD) to minimize inter-observer variability.

### Outcomes

The primary outcome was occurrence of in-hospital postoperative complications, defined as unexpected medical events occurring between the start of the operation and discharge from hospital. For the chart review, postoperative complications were stratified by severity using the Clavien Classification of Surgical Complications [18] system (See Additional file 1). Each patient was assigned a postoperative status (≥ 1 versus 0 complications) and severity by Clavien class (I-V). For patients experiencing more than one postoperative complication, the most severe complication class was assigned. Complications were also stratified by category: gastrointestinal, cardiovascular, infectious, etc. See Additional file 1: Table S2 for the specific complications comprising each category. In the administrative database, we identified postoperative complications based on pre-defined ICD-9 and CCI codes that have been commonly used to identify postoperative complications [11]. Complication codes used in the analysis can be found in Additional file 1: Table S3.

### Variables

Variables extracted from both chart review and administrative data included age at colectomy; emergent versus elective operation; reason for colectomy (UC refractory to medical management, dysplasia or cancer, and acute complication of UC); and pre-admission comorbidities defined by the Charlson-Deyo[19] and Elixhauser [20] indices. Comorbidities were stratified by activity status to identify medical conditions that were managed during the admission. Colectomies were documented as elective if the decision to operate was made prior to hospital admission; in contrast, the decision for emergent colectomy occurred during the admission (e.g. in response to acute life-threatening complications of UC flare or medically refractory disease). In the administrative database, elective colectomies were defined as those coded with an ‘elective’ status, while emergent colectomies were defined using a composite of either ‘emergent’ or ‘urgent’ codes. Age, comorbidity, and admission type were *a priori* defined as preoperative risk factors and subsequently validated because previous studies have shown that they were associated with postoperative complications in UC patients [11,21].

### Data analysis

The administrative coding was validated against the chart review for the study population, admission type, comorbidity status, and postoperative complications. In our primary cohort, we validated the accuracy of administrative data in identifying UC patients undergoing colectomy. Secondarily, a cohort of UC patients admitted for flare without operation was reviewed to detect colectomy patients not captured in our primary cohort. Sensitivity, specificity, positive predictive value (PPV), and negative predictive value (NPV) with 95% CIs were calculated for UC diagnostic codes alone and the combination of UC + colectomy codes. For patients presenting with UC flare, the analysis was also stratified by UC diagnostic position. In secondary analysis, we validated the accuracy of specific procedural colectomy codes. We also validated the accuracy of administrative data defining emergent or urgent versus elective colectomy. In a sensitivity analysis, we excluded patients admitted urgently, and compared only emergent versus elective patients.

The validity of administrative data in capturing comorbid conditions in colectomy patients was also assessed. We searched the administrative database to identify patients coded with Charlson-Deyo comorbidities and cross-matched these with the true comorbidities identified by chart review. Sensitivity, specificity, PPV, and NPV with 95% CIs were calculated for administrative data predicting whether patients had 0 versus ≥ 1 pre-hospital comorbidities and for each of the 17 specific comorbidities. Subgroup analysis of only comorbidities active on admission was performed. Analyses were repeated for Elixhauser comorbidities (Additional file 1: Table S4-S6.

We assessed the validity of administrative data in capturing in-hospital postoperative complications. Patients coded with complications in the administrative database were cross-matched with those identified by chart review. Sensitivity and specificity with 95% CIs was then calculated under 3 scenarios: 1) none versus any complications; 2) none or Clavien class I versus class II-V complications; and 3) none or class I-II versus class III-V complications. Sensitivity and specificity in capturing specific categories of complications was also determined.

Multivariate logistic regression analysis was performed to examine the association between preoperative risk factors and postoperative complication. In primary analysis, postoperative complication status was defined as none versus any complication. Age (defined as 18–34, 35–64, and ≥ 65 years), comorbidity (0 versus any Charlson comorbidity, and secondarily Elixhauser), and admission type (emergency versus elective) were *a priori* included into the logistic regression model. Odds ratios with 95% confidence intervals (CI) were calculated for each preoperative risk factor. Two logistic regression models were developed for comparison: 1) data derived from chart review and 2) data derived from the administrative database.

Statistical analyses were performed using SAS statistical software (version 9.2, SAS Institute Inc., Cary, NC). Ethics approval for the study protocol was granted by the Conjoint Health Research Ethics Board at the University of Calgary, study #21833.

## Results

### Study population

**Figure 1:**
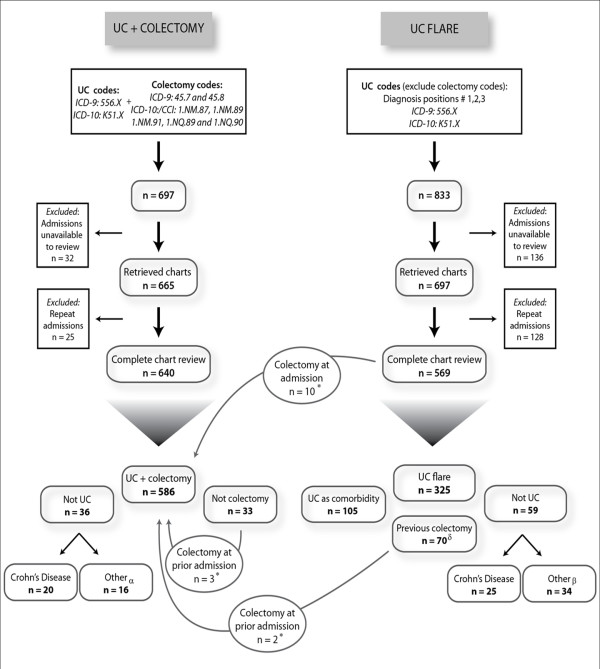
Approach to identifying UC patients who flared and those who underwent colectomy. α “Other Not UC”: Ischemic colitis n = 1, Pseudomembranous colitis n = 3, unspecified n = 12. β “Other Not UC”: Ischemic colitis n = 4, Pseudomembranous colitis n = 7, unspecified n = 23. δ “Previous colectomy”: Record of previous colectomy occurring before the study period 1996–2007 (n = 68), record of previous colectomy occurring in study period (n = 2). * Subset of colectomy patients (n = 15), not captured by administrative definition.

The cohort identification process is illustrated in Figure 1. Search of the administrative database from 1996 to 2007 for patients with UC and colectomy codes found 697 admissions. Excluding unavailable charts (n = 32) and repeated admissions for the same patient (n = 25), we completed chart review on 640 patients and identified 586 patients who underwent colectomy for UC. Among 586 patients, 60% were male and 53% underwent an elective operation. Median age at operation was 40.0 years [interquartile range: 30.0–53.0]. Thirty three percent of UC patients had at least one Charlson comorbidity and 39% experienced an in-hospital postoperative complication following colectomy.

For the non-operative UC flare cohort, 833 admissions were identified as having UC in diagnostic positions 1–3 with the exclusion of colectomy codes. Among these, 569 charts were reviewed; 136 charts were unavailable and128 were repeat admissions. We identified 325 patients who were admitted for a flare of their UC; of these, 298 (91.7%) had UC as the primary diagnosis.

### Validation of study population

The administrative database correctly identified both UC and colectomy in 85.9% [83.2–88.5%] of cases (571/665). PPV for specific colectomy codes is found in Table 1. Reasons for misclassification included: repeat admissions (n = 25, 3.8%); patients did not have UC (n = 36, 5.4%); and patients did not undergo colectomy (n = 33, 5.0%). Most commonly, patients were misclassified because the diagnosis was Crohn’s disease (n = 20), ischemic or pseudomembranous colitis (n = 4), or laparoscopic investigative studies rather than colectomy.

**Table 1:** Validation of Colectomy Procedural Codes

**Procedural codes**	**Definition**	**Total Codes (n)**	**Colectomy (n)**	**PPV** **(95% CI)**
45.7 or 45.8*	Open and other partial excision of large intestine or total intra-abdominal colectomy	294	275	0.94 [0.90–0.96]
**1.NM.87**	Excision partial, large intestine	45	20	0.44 [0.31–0.59]
**1.NM.89**	Excision total, large intestine endoscopic	52	47	0.90 [0.79–0.96]
**1.NM.91**	Excision radical, large intestine	1	1	1.00 [0.21–1.00]
**1.NQ.89 or 1.NQ.90**^**α**^	Excision total, rectum or excision total with reconstruction, rectum using open approach with ileum	273	228	0.84 [0.79–0.87]
**All colectomies**		**665**	**571**	**0.86** **[0.83**–**0.88]**

^*^ 1 chart identified coded with both 45.7 and 45.8 codes.

^α^ 3 charts identified coded with both 1.NQ.89 and 1.NQ.90 codes.

The administrative database was 94.4% [90.9%–96.6%] sensitive at identifying patients admitted for emergent colectomy; specificity was 76.4% [71.3%–80.8%]. Sensitivity analysis comparing only patients coded as ‘elective’ or ‘emergent’ (i.e. excluded patients admitted under ‘urgent’ status) showed improved specificity (96.3% [93.1%–98.0%]) but decreased sensitivity (85.5% [76.7%–90.7%]).

Searching the non-operative UC flare cohort identified an additional 15 colectomy patients who were missed in the primary search. Overall, the administrative data was 46.6% [42.9%–50.3%] accurate in identifying patients presenting with a flare of UC (325/697). Reasons for misclassification included: prior colectomy (n = 70); patients did not have UC (n = 59), or most commonly, UC was a comorbidity for an unrelated admission (n = 105). The accuracy of administrative data identifying a UC flare varied by diagnostic position: 79.3% [75.2%–83.4%] in the primary diagnostic position; 18.7% [11.3%–26.1%] in the second position; and 8.1% [2.4%–13.9%] in the third position.

### Outcome validation

Chart review determined that 39.2% (224/571) of patients undergoing colectomy experienced at least one postoperative complication and 12.3% (70/571) experienced at least one severe postoperative complication (Clavien class III or greater). Sensitivity of administrative data in identifying patients who experienced at least one postoperative complication was 68.3% [61.8%–74.3%] with specificity of 83.6% [79.3%–87.1%], PPV 72.9% [66.5%–78.4%], and NPV 80.3% [75.9%–84.1%]. Results stratified by complication category can be found in Table 2. With advancing complication severity, the sensitivity of the administrative database increased while the specificity decreased (Table 2). The administrative data was the most sensitive at identifying Clavien class III or greater postoperative complications (84.3% [73.6%–91.9%]); however, the specificity was reduced to 69.9% [65.6%–73.9%].

**Table 2:** Validation of postoperative complication coding in the administrative database. Sensitivity and specificity with 95% CI of administrative data in identifying postoperative complications stratified by complication group and severity (determined using Clavien Classification I-V)

**Complication group**	**Total**	**Sensitivity**	**Specificity**
**Any Complication**			
0 vs ≥1	224	0.68 [0.62 – 0.74]	0.84 [0.79 – 0.87]
0/Class I vs. Class II-V	154	0.78 [0.71 – 0.84]	0.78 [0.74 – 0.82]
0/Class I-II vs. Class III-V	70	0.84 [0.74 – 0.92]	0.70 [0.66 – 0.74]
**Gastrointestinal complications**			
0 vs ≥1	108	0.56 [0.47 – 0.66]	0.89 [0.85 – 0.91]
0/Class I vs. Class II-V	51	0.67 [0.52 – 0.79]	0.85 [0.81 – 0.88]
0/Class I-II vs. Class III-V	23	0.78 [0.56 – 0.93]	0.82 [0.79 – 0.86]
**Infections**			
0 vs ≥1	78	0.59 [0.47 – 0.70]	0.96 [0.93 – 0.97]
0/Class I vs. Class II-V	69	0.58 [0.45 – 0.70]	0.84 [0.92 – 0.96]
0/Class I-II vs. Class III-V	29	0.66 [0.46 – 0.82]	0.91 [0.88 – 0.93]
**Wounds**			
0 vs ≥1	38	0.50 [0.33 – 0.67]	0.96 [0.94 – 0.98]
0/Class I vs. Class II-V	31	0.48 [0.30 – 0.67]	0.96 [0.94 – 0.97]
0/Class I-II vs. Class III-V	30	0.47 [0.28 – 0.66]	0.96 [0.93 – 0.97]
**Renal and Endocrine complications**			
0 vs ≥1	32	0.66 [0.47 – 0.81]	0.93 [0.91 – 0.95]
0/Class I vs. Class II-V	27	0.74 [0.54 – 0.89]	0.93 [0.91 – 0.95]
0/Class I-II vs. Class III-V	6	1.00 [0.54 – 1.00]	0.91 [0.88 – 0.93]
**Cardiovascular disorders**			
0 vs ≥1	54	0.67 [0.53 – 0.79]	0.97 [0.96 – 0.99]
0/Class I vs. Class II-V	46	0.74 [0.59 – 0.86]	0.97 [0.95 – 0.98]
0/Class I-II vs. Class III-V	18	0.89 [0.65 – 0.99]	0.94 [0.92 – 0.96]
**Pulmonary complications**			
0 vs ≥1	31	0.42 [0.25 – 0.61]	0.97 [0.95 – 0.98]
0/Class I vs. Class II-V	19	0.53 [0.29 – 0.76]	0.96 [0.94 – 0.98]
0/Class I-II vs. Class III-V	14	0.71 [0.42 – 0.92]	0.96 [0.95 – 0.98]
**Neurological disorders**			
0 vs ≥1	12	0.42 [0.15 – 0.72]	0.99 [0.98 – 1.00]
0/Class I vs. Class II-V	6	0.67 [0.22 – 0.96]	0.99 [0.98 – 1.00]
0/Class I-II vs. Class III-V	1	1.00 [0.03 – 1.00]	0.99 [0.97 – 0.99]

### Comorbidity validation

Sensitivity of the administrative data in capturing patients with any Charlson comorbidities was 37.3% [30.8%–44.3%] with specificity 97.9% [95.9%–98.9%] (Tables 3 and 4). Sensitivity was improved when the analysis was restricted to active comorbidities alone (62.9% [51.1%–73.2%]); however specificity and PPV were reduced. Results stratified by specific comorbidity are presented in Tables 3 and 4. Overall PPV of the administrative database for identifying the presence of Charlson comorbidities was 90% [82%–95%]. Similar results for Elixhauser comorbidities are presented in Additional file 1: Table S4-S6.

**Table 3:** Validation of Charlson comorbidity coding in the administrative database. Sensitivity, specificity, PPV and NPV with 95% CI of administrative data in identifying preoperative Charlson comorbidities, stratified by comorbidity type

	**n**	**Sensitivity**	**Specificity**	**PPV**	**NPV**
Any Charlson comorbidity^*^					
Total^α^	**193**	**0.37 ** [0.31–0.44]	**0.98 ** [0.96–0.99]	**0.90 ** [0.82–0.95]	**0.75 ** [0.71–0.79]
Active at Admission^β^	**70**	**0.63 ** [0.51–0.73]	**0.93 ** [0.90–0.95]	**0.55 ** [0.44–0.65	**0.95 ** [0.92– 0.96]
**Any malignancy**					
Total	45	0.40	0.99	0.78	0.95
Active	24	0.75	0.99	0.78	0.99
**Cerebrovascular disease**					
Total	7	0.29	0.99	0.40	0.99
Active	0	N/A	0.99	0.00	1.00
**Chronic pulmonary disease**					
Total	70	0.21	1.00	0.88	0.90
Active	7	0.29	0.97	0.12	0.99
**Congestive heart failure**					
Total	13	0.31	0.99	0.50	0.98
Active	0	N/A	0.99	0.00	1.00
**Diabetes w/complication**					
Total	5	0.60	1.00	1.00	1.00
Active	2	0.50	1.00	0.33	1.00
**Diabetes w/o complication**					
Total	30	0.57	1.00	0.89	0.98
Active	14	0.43	0.98	0.32	0.99
**Hemiplegia/paraplegia**					
Total	2	0.00	1.00	0.00	1.00
Active	1	0.00	1.00	0.00	1.00

^α^ Denotes total number of patients with the specified comorbidity. 193 patients presented with at least 1 Charlson comorbidity. However, sum of patients with each comorbidity type does not equal 193 as patients may have > 1 Charlson comorbidity.

^β^ Comorbidities stratified in chart review by activity on admission.

^*^ No patients identified with AIDS or dementia.

**Table 4:** Validation of Charlson comorbidity coding in the administrative database continued. Sensitivity, specificity, PPV and NPV with 95% CI of administrative data in identifying preoperative Charlson comorbidities, stratified by comorbidity type

	**n**	**Sensitivity**	**Specificity**	**PPV**	**NPV**
**Mild liver disease**					
Total^α^	21	0.19	0.99	0.44	0.97
Active^β^	6	0.33	0.99	0.22	0.99
**Moderate/severe liver disease**					
Total	5	0.40	1.00	0.50	0.99
Active	4	0.50	1.00	0.50	1.00
**Myocardial infarction**					
Total	21	0.38	0.99	0.67	0.98
Active	1	1.00	0.98	0.08	1.00
**Peptic ulcer disease**					
Total	11	0.27	0.99	0.50	0.99
Active	3	1.00	0.99	0.50	1.00
**Peripheral vascular disease**					
Total	6	0.17	1.00	0.33	0.99
Active	1	0.00	0.99	0.00	1.00
**Renal disease**					
Total	8	0.13	1.00	0.50	0.99
Active	3	0.33	1.00	0.50	1.00
**Rheumatoid arthritis or collagen disease**					
Total	38	0.13	1.00	0.83	0.94
Active	12	0.17	0.99	0.33	0.98

^α^ Denotes total number of patients with the specified comorbidity. 193 patients presented with at least 1 Charlson comorbidity. However, sum of patients with each comorbidity type does not equal 193 as patients may have > 1 Charlson comorbidity.

^β^ Comorbidities stratified in chart review by activity on admission.

### Risk factors for postoperative complications – administrative versus chart review data

Both chart review and administrative data similarly predicted that age ≥ 65 increased the risk of postoperative complications by approximately 2-fold (Table 5). Although emergent admission status was a risk factor for postoperative complication, the odds ratio for emergent admission was higher in the administrative database (OR 2.52 [1.80–3.52]) than that in the chart review (OR 1.49 [1.06–2.09]). The odds ratio for presence of ≥1 Charlson comorbidity was also higher in administrative data (OR 2.91 [1.86–4.56]) as compared to chart data (OR 1.50 [1.05–2.15]) (Table 5).

**Table 5:** Risk factors for postoperative complications following colectomy in UC patients defined by chart review vs. administrative data

**Risk Factor**	Chart Data OR (95%CI) n = 586*	Administrative Data OR (95%CI) n = 697*
**Age**		
18–34	1.0	1.0
35–64	0.87 (0.60–1.26)	0.83 (0.58–1.19)
65+	1.97 (1.10–3.52)	2.04 (1.18–3.52)
**Admission Type**		
Elective	1.0	1.0
Emergent	1.49 (1.06–2.09)	2.52 (1.80–3.52)
**Charlson Comorbidity**		
0	1.0	1.0
≥ 1	1.50 (1.05–2.15)	2.91 (1.86–4.56)

OR - odds ratios; CI - confidence interval.

^*^ Cohort of patients identified by administrative data (n = 697) includes all patients coded with UC + colectomy. The cohort used for chart data analysis excludes repeated admissions, patients without UC or colectomy, and unavailable charts (See Figure 1).

## Discussion

We conducted this study to evaluate whether outcomes derived from administrative databases accurately represent outcomes obtained from retrospectively reviewing medical charts. Both the administrative database and the chart review identified age, preoperative comorbidities, and emergent surgery as risk factors for postoperative complications following colectomy for UC. However, administrative data overestimated the magnitude of the risk for comorbidities and emergent operations as compared to chart review. Differences in risk estimates were in part explained by misclassification errors associated with the administrative database defining the study population, preoperative risk factors (i.e. comorbidity and emergent colectomy) and postoperative outcome (i.e. complications). The administrative database was more accurate at identifying comorbidities active at admission and the most severe postoperative complications; this selective coding likely biased the risk estimates away from the null hypothesis.

Both clinical [22,23] and administrative database studies [11,24] have identified advancing age, comorbidities, and emergency operations as risk factors for postoperative complications following colectomy and other abdominal surgeries [25-28], though few have evaluated the difference between the two methods[29]. In our analysis both the administrative and chart review data predicted an approximately two-fold increase in complications in those aged ≥ 65 compared to age 18–34 years. Close agreement of OR between administrative and chart data was expected because age is objective and reported with near perfect accuracy in both data sets.

The magnitude of effect for emergent operations was greater with the administrative data as compared to chart review. The administrative database was less specific for identifying emergent colectomy, with a high prevalence of false positives. Sensitivity analysis excluding patients with ‘urgent’ codes demonstrated improved specificity suggesting that the code ‘urgent’ is more aligned with an elective, rather than emergent admission. For example, patients electively admitted for an operation occurring within 24 hours of admission were at times coded as ‘urgent’.

Adaptations of the Charlson and Elixhauser comorbidity indices [20,30] have been validated for risk adjustment of postoperative morbidity and mortality [31-33]. In our analysis, Charlson comorbidities were significantly associated with worse postoperative outcomes; though, the magnitude of effect was greater in the administrative database than chart data. Preferential recording of comorbidities actively managed in-hospital may explain this difference. The sensitivity for most comorbid illnesses was low, but increased when the analysis was restricted to active comorbidities. Our findings were similar to other validation studies that have found poor sensitivity of comorbidity coding[15] and underreporting of chronic comorbidities not requiring treatment [34]. Despite the low sensitivity of administrative data, other studies have found that prediction of in-hospital mortality was identical to indices derived from chart review [31,35]. Additionally, among patients with multiple postoperative complications physicians may record more comorbidities in the discharge summary to explain the poor outcomes, while this may not be detailed in patients with an uncomplicated postoperative recovery.

The administrative database was 86% accurate in identifying patients with UC undergoing colectomy. Additionally, a small subset (n = 15) of UC patients who underwent colectomy were not recorded in the administrative database. Thirumurthi *et al.* found the sensitivity of the diagnostic code 556 × for hospitalization of UC was 84% [36]. Diagnostic coding for UC may be less accurate than for other conditions; for instance, validations of administrative data in patients presenting with heart failure, acute COPD exacerbations, acute coronary syndromes, and subarachnoid haemorrhage have consistently demonstrated PPV of diagnostic codes exceeding 95% [37-40]. In UC, the lower PPV may reflect uncertainties in diagnosis, especially from Crohn’s disease and other causes of colitis. A previous study also reported higher PPV (96.1%) for colectomy codes[41] compared to our findings, although that validation was performed in a cohort of general surgery patients, with a smaller sample size (n = 56), and included procedural codes for rectal resections (484, 485, 486). In our study, follow-up procedures such as second stage ileopouch anal anastomosis were commonly misclassified as colectomies.

Administrative data did not reliably identify UC patients admitted with a flare without colectomy when the first three diagnostic positions were searched. Although nearly 80% of admissions with UC coded in the primary diagnostic position represented an acute flare of disease, UC recorded in the second or third diagnostic positions represented an acute flare in fewer than 10% of cases. This misclassification error is evident in the literature, as one study demonstrated strengthening of risk estimates when a sensitivity analysis was conducted to exclude Crohn’s disease patients admitted to hospital with a secondary diagnosis of Crohn’s disease [42]. Consequently, prior studies using administrative databases have likely overestimated the true hospitalization rate of UC patients admitted for an acute flare of disease when non-primary diagnostic positions were searched.

The validity of postoperative complications in UC has not been reported. In our study, administrative data was 68% sensitive in identifying patients experiencing at least one complication after colectomy. Previous studies have also shown underreporting of complications in administrative data [43-47]. Misclassification of postoperative complications contributed to the discrepancy observed between administrative and chart review data. The accuracy of administrative data in coding postoperative complications was correlated to complication severity: sensitivity increased when less severe complications were excluded from the analysis while the specificity decreased. Administrative data poorly identified minor complications (i.e. Clavien I), but captured the more severe and clinically significant postoperative complications. These findings were similar to our comorbidity validation, supporting the notion that administrative databases miss comorbidities and complications that likely have less clinical impact.

Misclassification of post-operative complications was predominantly due to the challenge in differentiating a postoperative complication from a comorbidity or a preoperative in-hospital complication. For example, UC patients who underwent colectomy and were coded for pulmonary embolism were recorded as a false positive if the pulmonary embolism was diagnosed before the colectomy was performed.

Several limitations of our study should be considered. First, the chart review was retrospective and not all clinical information may have been documented in the charts. As we comprehensively reviewed only the current admission, other comorbidities may have been missed. Second, we only had access to administrative codes for the patient’s hospitalization for colectomy; searching prior admissions may have improved the sensitivity of administrative coding, particularly for comorbidities. This provides an area for future study that may be explored in other datasets. Third, variation between reviewers was unavoidable although we attempted to limit inter-observer variability. Fourth, a small portion (2.6%) of UC patients coded for a flare but not colectomy actually underwent colectomy when the chart was reviewed. Conceivably, UC patients who underwent colectomy may not have been coded for either UC or colectomy, though this misclassification error is likely far less than 2.6%. Fifth, our sample size was sufficient to evaluate the overall validity of administrative data, but uncommon comorbidities and complications could not be validated. Similarly, large administrative database studies have the power to stratify comorbidity as a categorical variable (i.e. 0, 1, 2, or ≥3 comorbidities), but the prevalence of multiple comorbidities in our cohort was too low to accurately perform this subgroup analysis. Finally, the administrative database reflects the quality associated with Calgary’s DAD and thus, may not be generalized to other hospitalization databases. However, Calgary’s DAD is comprehensive, has been widely used and validated for health service research[41], and has demonstrated generalizability in different settings. For example, a recent study demonstrated that Charlson comorbidities predicted in-hospital mortality similarly in Calgary’s hospital DAD as compared to hospitalization databases in France, New Zealand, Japan, Switzerland, and Australia [48]. Thus, the data from this study should reflect practices and outcomes of other administrative databases and at minimum should motivate others to test the validity of local administrative databases.

## Conclusions

Administrative data identified the same risk factors (advanced age, emergency admission, and comorbidities) for postoperative complications as chart review. However, the risk estimates were biased away from the null by the administrative database. The discrepancy in risk estimates may be explained by inaccuracies in defining the study population, complications, and comorbidities. Administrative data more accurately identified severe postoperative complications and comorbidities actively managed during the admission. Thus, despite the imperfect validity of administrative data, identified comorbidities and complications were likely the most clinically meaningful. Administrative databases are valid tools for IBD research, but the general inferences drawn from risk estimates should be interpreted in the context of limitations associated in identifying the study population, risk factors, and postoperative complications.

## Competing interests

The authors declare that they have no competing interests.

## Author contributions

All authors have reviewed and approved this manuscript. Study design and planning, data interpretation, manuscript drafting and approval: Dr. GK. Study planning, data collection and interpretation, manuscript drafting and approval: CMa. Study planning, data collection, manuscript approval: MC, M-CP, and MP. Data analysis and manuscript approval: JH. Study planning, data collection and interpretation, manuscript approval: Dr. SDS. Manuscript drafting and approval: Dr. RP, Dr. SG, Dr. RM, and Dr. HQ.

## Pre-publication history

The pre-publication history for this paper can be accessed here:

http://www.biomedcentral.com/1471-230X/12/39/prepub

## Supplementary Material

Additional file 1 **Tables S1-S6.**For Table S1, please see reference citation number [18], Dindo 2004. Click here for file

## Acknolwedgements

We acknowledge Andrew Fong for providing data from the Calgary Health Zone. Christopher Ma was supported in part by a summer studentship award from the Alberta Heritage Foundation for Medical Research (AHFMR). Martin Prusinkiewicz was supported by an American Gastroenterological Association Foundation Student Research Fellowship Award. Dr. Kaplan is supported through a New Investigator Award from the Canadian Institute of Health Research (CIHR) and a Population Health Investigator Award from the AHFMR. Dr. Quan was supported by AHFMR Health Scholar award. Dr. Myers is supported by a New Investigator Award from the CIHR and a Population Health Investigator Award from the AHFMR. The M.S.I. Foundation provided funding for this study.

Funding Support M.S.I. Foundation

## References

[B1] Solberg IC Lygren I Jahnsen J Aadland E Hoie O Cvancarova M Bernklev T Henriksen M Sauar J Vatn MH Clinical course during the first 10 years of ulcerative colitis: results from a population-based inception cohort (IBSEN Study) Scand J Gastroenterol 2009 44 4 431 440 10.1080/00365520802600961 19101844

[B2] Cima RR Pemberton JH Medical and surgical management of chronic ulcerative colitis Arch Surg 2005 140 3 300 310 10.1001/archsurg.140.3.300 15781797

[B3] Shen B Remzi FH Lavery IC Lashner BA Fazio VW A proposed classification of ileal pouch disorders and associated complications after restorative proctocolectomy Clin Gastroenterol Hepatol 2008 6 2 145 158 quiz 124 10.1016/j.cgh.2007.11.006 18237865

[B4] Fazio VW Ziv Y Church JM Oakley JR Lavery IC Milsom JW Schroeder TK Ileal pouch-anal anastomoses complications and function in 1005 patients Ann Surg 1995 222 2 120 127 10.1097/00000658-199508000-00003 7639579 PMC1234769

[B5] Meagher AP Farouk R Dozois RR Kelly KA Pemberton JH J ileal pouch-anal anastomosis for chronic ulcerative colitis: complications and long-term outcome in 1310 patients Br J Surg 1998 85 6 800 803 10.1046/j.1365-2168.1998.00689.x 9667712

[B6] Dayton MT Larsen KP Outcome of pouch-related complications after ileal pouch-anal anastomosis Am J Surg 1997 174 6 728 731 discussion 731–722 10.1016/S0002-9610(97)00188-8 9409606

[B7] Romanos J Samarasekera DN Stebbing JF Jewell DP Kettlewell MG Mortensen NJ Outcome of 200 restorative proctocolectomy operations: the John Radcliffe Hospital experience Br J Surg 1997 84 6 814 818 10.1002/bjs.1800840623 9189096

[B8] Alves A Panis Y Bouhnik Y Maylin V Lavergne-Slove A Valleur P Subtotal colectomy for severe acute colitis: a 20-year experience of a tertiary care center with an aggressive and early surgical policy J Am Coll Surg 2003 197 3 379 385 10.1016/S1072-7515(03)00434-4 12946792

[B9] Pal S Sahni P Pande GK Acharya SK Chattopadhyay TK Outcome following emergency surgery for refractory severe ulcerative colitis in a tertiary care centre in India BMC Gastroenterol 2005 5 39 10.1186/1471-230X-5-39 16316474 PMC1325033

[B10] Mikkola KA Jarvinen HJ Management of fulminating ulcerative colitis Ann Chir Gynaecol 1992 81 1 37 41 1622050

[B11] Kaplan GG McCarthy EP Ayanian JZ Korzenik J Hodin R Sands BE Impact of hospital volume on postoperative morbidity and mortality following a colectomy for ulcerative colitis Gastroenterology 2008 134 3 680 687 10.1053/j.gastro.2008.01.004 18242604

[B12] De Coster C Quan H Finlayson A Gao M Halfon P Humphries KH Johansen H Lix LM Luthi JC Ma J Identifying priorities in methodological research using ICD-9-CM and ICD-10 administrative data: report from an international consortium BMC Health Serv Res 2006 6 77 10.1186/1472-6963-6-77 16776836 PMC1513221

[B13] Ananthakrishnan AN McGinley EL Binion DG Does it matter where you are hospitalized for inflammatory bowel disease? A nationwide analysis of hospital volume Am J Gastroenterol 2008 103 11 2789 2798 10.1111/j.1572-0241.2008.02054.x 18684184

[B14] Nguyen GC Laveist TA Gearhart S Bayless TM Brant SR Racial and geographic variations in colectomy rates among hospitalized ulcerative colitis patients Clin Gastroenterol Hepatol 2006 4 12 1507 1513 10.1016/j.cgh.2006.09.026 17162242

[B15] Preen DB Holman CD Lawrence DM Baynham NJ Semmens JB Hospital chart review provided more accurate comorbidity information than data from a general practitioner survey or an administrative database J Clin Epidemiol 2004 57 12 1295 1304 10.1016/j.jclinepi.2004.03.016 15617956

[B16] Kaplan GG Administrative database studies in IBD: a cautionary tale Am J Gastroenterol 2010 105 8 1808 1810 10.1038/ajg.2010.232 20686467

[B17] Alberta Health Services Annual Report, April 1, 2009 - March 31, 2010 [http://www.albertahealthservices.ca/Publications/ahs-pub-annual-rpt.pdf]

[B18] Dindo D Demartines N Clavien PA Classification of surgical complications: a new proposal with evaluation in a cohort of 6336 patients and results of a survey Ann Surg 2004 240 2 205 213 10.1097/01.sla.0000133083.54934.ae 15273542 PMC1360123

[B19] Deyo RA Cherkin DC Ciol MA Adapting a clinical comorbidity index for use with ICD-9-CM administrative databases J Clin Epidemiol 1992 45 6 613 619 10.1016/0895-4356(92)90133-8 1607900

[B20] Elixhauser A Steiner C Harris DR Coffey RM Comorbidity measures for use with administrative data Med Care 1998 36 1 8 27 10.1097/00005650-199801000-00004 9431328

[B21] de Silva S Ma C Proulx MC Crespin M Kaplan BS Hubbard J Prusinkiewicz M Fong A Panaccione R Ghosh S Postoperative complications and mortality following colectomy for ulcerative colitis Clin Gastroenterol Hepatol 2011 9 11 972 980 10.1016/j.cgh.2011.07.016 21806954

[B22] Bender JS Bouwman DL Total abdominal colectomy: conditions defining outcome Am Surg 1994 60 3 205 209 8116983

[B23] Costa G Tomassini F Tierno SM Venturini L Frezza B Cancrini G Mero A Lepre L Emergency colonic surgery: analysis of risk factors predicting morbidity and mortality Chir Ital 2009 61 5–6 565 571 20380259

[B24] Longo WE Virgo KS Johnson FE Oprian CA Vernava AM Wade TP Phelan MA Henderson WG Daley J Khuri SF Risk factors for morbidity and mortality after colectomy for colon cancer Dis Colon Rectum 2000 43 1 83 91 10.1007/BF02237249 10813129

[B25] Ondrula DP Nelson RL Prasad ML Coyle BW Abcarian H Multifactorial index of preoperative risk factors in colon resections Dis Colon Rectum 1992 35 2 117 122 10.1007/BF02050665 1735312

[B26] Massarweh NN Legner VJ Symons RG McCormick WC Flum DR Impact of advancing age on abdominal surgical outcomes Arch Surg 2009 144 12 1108 1114 10.1001/archsurg.2009.204 20026827

[B27] Tan PY Stephens JH Rieger NA Hewett PJ Laparoscopically assisted colectomy: a study of risk factors and predictors of open conversion Surg Endosc 2008 22 7 1708 1714 10.1007/s00464-007-9702-1 18071801

[B28] Antolovic D Koch M Hinz U Schottler D Schmidt T Heger U Schmidt J Buchler MW Weitz J Ischemic colitis: analysis of risk factors for postoperative mortality Langenbecks Arch Surg 2008 393 4 507 512 10.1007/s00423-008-0300-z 18286300

[B29] Jang SH Chea JW Lee KB Charlson comorbidity index using administrative database in incident PD patients Clin Nephrol 2010 73 3 204 209 20178719 10.5414/cnp73204

[B30] Needham DM Scales DC Laupacis A Pronovost PJ A systematic review of the Charlson comorbidity index using Canadian administrative databases: a perspective on risk adjustment in critical care research J Crit Care 2005 20 1 12 19 10.1016/j.jcrc.2004.09.007 16015512

[B31] Quan H Parsons GA Ghali WA Validity of information on comorbidity derived from ICD-9-CCM administrative data Med Care 2002 40 8 675 685 10.1097/00005650-200208000-00007 12187181

[B32] Sundararajan V Henderson T Perry C Muggivan A Quan H Ghali WA New ICD-10 version of the Charlson comorbidity index predicted in-hospital mortality J Clin Epidemiol 2004 57 12 1288 1294 10.1016/j.jclinepi.2004.03.012 15617955

[B33] Sundararajan V Quan H Halfon P Fushimi K Luthi JC Burnand B Ghali WA Cross-national comparative performance of three versions of the ICD-10 Charlson index Med Care 2007 45 12 1210 1215 10.1097/MLR.0b013e3181484347 18007172

[B34] Erler A Beyer M Muth C Gerlach FM Brennecke R Garbage in - garbage out? Validity of coded diagnoses from GP claims records Gesundheitswesen 2009 71 12 823 831 10.1055/s-0029-1214399 19387933

[B35] Humphries KH Rankin JM Carere RG Buller CE Kiely FM Spinelli JJ Co-morbidity data in outcomes research: are clinical data derived from administrative databases a reliable alternative to chart review? J Clin Epidemiol 2000 53 4 343 349 10.1016/S0895-4356(99)00188-2 10785564

[B36] Thirumurthi S Chowdhury R Richardson P Abraham NS Validation of ICD-9-CM Diagnostic Codes for Inflammatory Bowel Disease Among Veterans Dig Dis Sci 2009 10.1007/s10620-009-1074-z 20033847

[B37] Lee DS Donovan L Austin PC Gong Y Liu PP Rouleau JL Tu JV Comparison of coding of heart failure and comorbidities in administrative and clinical data for use in outcomes research Med Care 2005 43 2 182 188 10.1097/00005650-200502000-00012 15655432

[B38] Ginde AA Blanc PG Lieberman RM Camargo CA Validation of ICD-9-CM coding algorithm for improved identification of hypoglycemia visits BMC Endocr Disord 2008 8 4 10.1186/1472-6823-8-4 18380903 PMC2323001

[B39] Varas-Lorenzo C Castellsague J Stang MR Tomas L Aguado J Perez-Gutthann S Positive predictive value of ICD-9 codes 410 and 411 in the identification of cases of acute coronary syndromes in the Saskatchewan Hospital automated database Pharmacoepidemiol Drug Saf 2008 17 8 842 852 10.1002/pds.1619 18498081

[B40] Kirkman MA Mahattanakul W Gregson BA Mendelow AD The accuracy of hospital discharge coding for hemorrhagic stroke Acta Neurol Belg 2009 109 2 114 119 19681442

[B41] Quan H Parsons GA Ghali WA Validity of procedure codes in International Classification of Diseases, 9th revision, clinical modification administrative data Med Care 2004 42 8 801 809 10.1097/01.mlr.0000132391.59713.0d 15258482

[B42] Ananthakrishnan AN McGinley EL Binion DG Saeian K A novel risk score to stratify severity of Crohn's disease hospitalizations Am J Gastroenterol 2010 105 8 1799 1807 10.1038/ajg.2010.105 20216534

[B43] Quan H Parsons GA Ghali WA Assessing accuracy of diagnosis-type indicators for flagging complications in administrative data J Clin Epidemiol 2004 57 4 366 372 10.1016/j.jclinepi.2003.01.002 15135837

[B44] Hartz AJ Kuhn EM Comparing hospitals that perform coronary artery bypass surgery: the effect of outcome measures and data sources Am J Public Health 1994 84 10 1609 1614 10.2105/AJPH.84.10.1609 7943479 PMC1615096

[B45] Newton KM Wagner EH Ramsey SD McCulloch D Evans R Sandhu N Davis C The use of automated data to identify complications and comorbidities of diabetes: a validation study J Clin Epidemiol 1999 52 3 199 207 10.1016/S0895-4356(98)00161-9 10210237

[B46] Romano PS Chan BK Schembri ME Rainwater JA Can administrative data be used to compare postoperative complication rates across hospitals? Med Care 2002 40 10 856 867 10.1097/00005650-200210000-00004 12395020

[B47] Geraci JM Ashton CM Kuykendall DH Johnson ML Wu L International Classification of Diseases, 9th Revision, Clinical Modification codes in discharge abstracts are poor measures of complication occurrence in medical inpatients Med Care 1997 35 6 589 602 10.1097/00005650-199706000-00005 9191704

[B48] Quan H Li B Couris CM Fushimi K Graham P Hider P Januel JM Sundararajan V Updating and validating the Charlson comorbidity index and score for risk adjustment in hospital discharge abstracts using data from 6 countries Am J Epidemiol 2011 173 6 676 682 10.1093/aje/kwq433 21330339

