# Supplementary material for: Postoperative complications following colectomy for ulcerative colitis: A validation study
Source: BMC Gastroenterol. 2012 Apr 27;12:39. doi: 10.1186/1471-230X-12-39 (PMC3432603; doi:10.1186/1471-230X-12-39)
Supplement: Additional file 1 — Tables S1-S6.For Table S1, please see reference citation number [18], Dindo 2004. [file 1471-230X-12-39-S1.doc]

Supplement Table 1: Clavien Classification[1] of Postoperative Complications

| **Grade** | **Definition** |
| --- | --- |
| **I** | Any deviation from normal postoperative course without need for pharmacological treatment or surgical, endoscopic, and radiological interventions  Allowed therapeutic regimens are: drugs as antiemetics, antipyretics, diuretics, electrolytes, and physiotherapy.  Wound infections opened at the bedside |
| **II** | Requiring pharmacological treatment with drugs other than such allowed for grade I complications  Blood transfusions and total parenteral nutrition |
| **III** | Requiring surgical, endoscopic, or radiological intervention |
| **IIIa** | Intervention not under general anesthesia |
| **IIIb** | Intervention under general anesthesia |
| **IV** | Life-threatening complication (including CNS complications) requiring intensive care/ICU management |
| **IVa** | Single organ dysfunction (including dialysis) |
| **IVb** | Multiorgan dysfunction |
| **V** | Death of a patient |

Supplement Table 2: Complication classification

| **Category** | **Complications** |
| --- | --- |
| **Gastrointestinal complications** | Small bowel obstruction, Anastomotic stricture (include peritoneal adhesions), Pouch leak Pouch failure, Bowel perforation, Ileus, Ischaemic bowel, GI bleeding (also include other hemorrhage and hemorrhagic conditions), Ileostomy / colostomy complication or malfunction, Digestive organ disorders (include acute hepatic failure and acute pancreatitis), Other GI complications (include pneumatosis) |
| **Wounds** | Fistula, Hematoma/seroma, Wound dehiscence and Delayed wound healing, Iatrogenic injuries (include foreign body accidentally left during procedure) and pressure ulcer. |
| **Infections** | Sepsis and bacteremia, Abscess, Wound infection, Urinary tract infection, Pneumonia and empyema, Other infections (include peritonitis, bacterial, skin and subcutaneous tissue infection). |
| **Renal and Endocrine complications** | Acute renal failure, Fluid and electrolyte disorders (include hypokalemia), Severe endocrine disorders (include adrenal disorders, hypoglycemic coma), Retention of urine (include atony of bladder), Other urinary complication (include urinary obstruction) |
| **Cardiovascular disorders** | Thrombosis/embolism, Myocardial infarction, Cardiac arrest, Hypotension or shock, Cardiac arrhythmias (exclude tachycardia), Heart failure, Other Cardiovascular complication ( include atherosclerotic heart disease, angina) |
| **Pulmonary complications** | Acute respiratory failure, Hypoxia, Pleural effusion and pulmonary edema, Pneumothorax and atelectasis, Other pulmonary complications (include asthma, extubation failure, difficulty breathing) |
| **Neurological disorders** | Cerebrovascular disease, Neurological disorders (psychoses/delirium/seizure), Disorders /complications of nervous system (include neuropathies) |

Supplement Table 3: ICD-9 and ICD-10 Complication Codes

| **Category** | **ICD-9** | **ICD-10** |
| --- | --- | --- |
| **Gastrointestinal** | 530.82, 531.0, 531.2, 531.4, 531.6, 532.0, 532.2, 532.4, 532.50, 532.6, 533.0, 533.2, 533.4, 533.6, 534.0, 534.2, 534.4, 534.6, 535.01, 535.11, 535.21, 535.31, 535.41, 535.51, 535.61, 557.0, 557.9, 560.1, 560.8, 560.81, 560.9, 568.0, 569.3, 569.60, 569.83, 569.89, 570, 577.0, 578, 997.4, 998.11, 998.89 | K22.8, K25.0, K25.2, K25.4, K25.6, K26.0, K26.1, K26.2, K26.4, K26.5, K26.6, K27.0, K27.2, K27.4, K27.6, K28.0, K28.2, K28.4, K28.6, K29.0, K55.0, K55.9, K56.0, K56.5, K56.6, K56.7, K62.5, K63.1, K63.8, K66.0, K65.5, K65.6, K72.0, K72.9 ,K85, K91.3, K91.4, K91.8, K91.9, K92, T79.2, T81.0, T88.8 |
| **Wounds** | 54.12, 54.61, 565.1, 569.69, 569.81, 575.4, 576.3, 599.1, 619.1, 707, 998.13, 998.12, 998.2, 998.3, 998.4, 998.83, 998.6, E870, E871 | K60.3, K60.4, K60.5, K63.2, K82.9, K83.2, L89, N36.0, N82.4, T81.2, T81.3, T81.5, T81.8, 1.OT.52.DA, 1.OT.56.DA, 1.OT.70.LA ,1.OW.80, 2.OT.70.LA |
| **Infections** | 038, 041, 480, 481, 482, 483, 484, 485, 486, 487.0, 507.0, 510, 513, 997.31, 997.39, 566, 567, 569.5, 569.61, 590.1, 590.2, 590.8, 590.9, 595.0, 595.9, 599.0, 682, 683, 790.7, 958.3, 996.6, 996.64, 998.5, 998.59, 998.7, 999.3 | A40, A41, A49, B95, B96, J10.0, J11.0, J12, J13, J14, J15, J16, J17, J18, J69.0, J85, J86, K61, K63.0, K65, L03, L04, N10, N12, N15.1, N15.9, N30.0, N30.9, N39.0, R78.8, T79.3, T80.2, T81.4, T81.6, T82.7, T83.6, T85.7 |
| **Renal and Endocrine** | 251.0, 255.4, 276, 584, 586, 596.4, 599.6, 788.2, 997.5 | E15, E272, E86, E87, N13.9, N17, N19, N31.2 ,N99.0, N99.9, R33 |
| **Cardiovascular disorders** | 410, 415.1, 427.3, 427.4, 427.5, 427.8, 427.9, 428, 444, 445, 449, 451, 452, 453, 458.2, 458.8, 458.9, 785.5, 958.0, 995.4, 997.1, 997.2, 997.7, 998.0, 999.1, 999.2 | I21, I26, I46, I48, I49, I50, I74, I80, I81, I82, I95.0, I95.2, I95.9, I97.8, I97.9, R57, T79.0, T80.0, T80.1, T81.1, T81.7, T88.2 |
| **Pulmonary** | 511.9, 512, 514, 518.0, 518.4, 518.5, 518.7, 518.81, 518.82, 799.0, 997.3 | J80, J81, J90, J91, J93, J95.5, J95.8, J95.9, J96.0, J96.9, J98.1, R09 |
| **Neurological disorders** | 292.1, 292.2, 292.81, 293, 342, 344, 348.1, 348.5, 349.0, 349.81, 349.9, 433, 434, 435, 436, 997.0, 997.00, 997.01, 997.02, 997.09 | F05, F13, F15, F19, G45, G46, G81, G82, G83, G93.1, G93.6, G97.0, G97.1, G97.8, G97.9, I63, I65 |

**Supplement Table 4: Validation of Elixhauser comorbidity coding in the administrative database.** Sensitivity, specificity, PPV and NPV with 95% CI of administrative data in identifying preoperative Elixhauser comorbidities, stratified by comorbidity type.

|  | **n** | **Sensitivity** | **Specificity** | **PPV** | **NPV** |
| --- | --- | --- | --- | --- | --- |
| **Any Elixhauser**  **comorbidity***  Total^α^  Active at Admission^β^ | **429**  **320** | **0.46**  [0.41-0.51]  **0.48**  [0.42-0.53] | **0.82**  [0.75-0.87]  **0.72**  [0.66-0.77] | **0.88**  [0.84-0.92]  **0.68**  [0.62-0.74] | **0.33**  [0.29-0.38]  **0.52**  [0.47-0.57] |
| **Alcohol Abuse**  Total  Active | 11  4 | 0.18  0.25 | 1.00  1.00 | 0.67  0.33 | 0.98  0.99 |
| **Blood Loss Anemia**  Total  Active | 47  43 | 0.17  0.16 | 0.99  0.99 | 0.62  0.54 | 0.93  0.94 |
| **Cardiac Arrhythmia**  Total  Active | 16  6 | 0.25  0.50 | 0.97  0.97 | 0.20  0.15 | 0.98  0.99 |
| **Chronic Pulmonary Disease**  Total  Active | 72  7 | 0.21  0.29 | 1.00  0.97 | 0.88  0.12 | 0.90  0.99 |
| **Coagulopathy**  Total  Active | 10  3 | 0.40  0.33 | 0.99  0.99 | 0.44  0.11 | 0.99  1.00 |
| **Congestive Heart Failure**  Total  Active | 12  0 | 0.33  N/A | 0.99  0.99 | 0.50  0.00 | 0.99  1.00 |
| **Deficiency Anemia**  Total  Active | 45  44 | 0.07  0.11 | 0.99  1.00 | 0.50  0.83 | 0.93  0.93 |

^α^ Denotes total number of patients with the specified comorbidity. 429 patients presented with at least 1 Elixhauser comorbidity. However, sum of patients with each comorbidity type does not equal 429 as patients may have > 1 Elixhauser comorbidity.

^β^ Comorbidities stratified in chart review by activity on admission. Active comorbidities are those requiring specific management at the time of admission.

^*^ No patients identified with comorbid AIDS, lymphoma, psychoses, or paralysis.

**Supplement Table 5: Validation of Elixhauser comorbidity coding in the administrative database continued.** Sensitivity, specificity, PPV and NPV with 95% CI of administrative data in identifying preoperative Elixhauser comorbidities, stratified by comorbidity type.

|  | **n** | **Sensitivity** | **Specificity** | **PPV** | **NPV** |
| --- | --- | --- | --- | --- | --- |
| **Depression**  **Total^α^**  **Active^β^** | 57  14 | 0.14  0.21 | 1.00  0.99 | 0.89  0.33 | 0.91  0.98 |
| **Diabetes Complicated**  **Total**  **Active** | 5  2 | 0.60  0.50 | 1.00  1.00 | 1.00  0.33 | 1.00  1.00 |
| **Diabetes Uncomplicated**  **Total**  **Active** | 29  13 | 0.59  0.46 | 1.00  0.98 | 0.89  0.32 | 0.98  0.99 |
| **Drug Abuse**  **Total**  **Active** | 16  10 | 0.06  0.00 | 0.99  0.99 | 0.20  0.00 | 0.97  0.98 |
| **Fluid/Electrolyte Disorders**  **Total**  **Active** | 50  38 | 0.48  0.53 | 0.85  0.84 | 0.23  0.19 | 0.94  0.96 |
| **Hypertension**  **Total**  **Active** | 60  6 | 0.57  0.83 | 0.99  0.94 | 0.89  0.13 | 0.95  1.00 |
| **Hypothyroidism**  **Total**  **Active** | 16  3 | 0.44  0.67 | 1.00  0.99 | 0.88  0.25 | 0.98  1.00 |
| **Liver Disease**  **Total**  **Active** | 27  10 | 0.26  0.40 | 0.99  0.99 | 0.64  0.36 | 0.96  0.99 |
| **Metastatic Cancer**  **Total**  **Active** | 3  1 | 1.00  1.00 | 0.99  0.99 | 0.33  0.11 | 1.00  1.00 |

^α^ Denotes total number of patients with the specified comorbidity. 429 patients presented with at least 1 Elixhauser comorbidity. However, sum of patients with each comorbidity type does not equal 429 as patients may have > 1 Elixhauser comorbidity.

^β^ Comorbidities stratified in chart review by activity on admission. Active comorbidities are those requiring specific management at the time of admission.

^*^ No patients identified with comorbid AIDS, lymphoma, psychoses, or paralysis.

**Supplement Table 6: Validation of Elixhauser comorbidity coding in the administrative database continued.** Sensitivity, specificity, PPV and NPV with 95% CI of administrative data in identifying preoperative Elixhauser comorbidities, stratified by comorbidity type.

|  | **n** | **Sensitivity** | **Specificity** | **PPV** | **NPV** |
| --- | --- | --- | --- | --- | --- |
| **Obesity**  **Total ^α^**  **Active ^β^** | 40  34 | 0.13  0.09 | 1.00  0.99 | 0.83  0.50 | 0.94  0.95 |
| **Other Neurological**  **Total**  **Active** | 12  1 | 0.25  0.00 | 0.99  0.99 | 0.43  0.00 | 0.98  1.00 |
| **Peptic Ulcer Disease**  **Total**  **Active** | 11  3 | 0.09  0.33 | 1.00  1.00 | 0.33  0.33 | 0.98  1.00 |
| **Peripheral Vascular Disease**  **Total**  **Active** | 6  1 | 0.17  0.00 | 1.00  0.99 | 0.33  0.00 | 0.99  1.00 |
| **Pulmonary Circulation Disorders**  **Total**  **Active** | 23  3 | 0.22  0.33 | 0.97  0.96 | 0.23  0.05 | 0.97  1.00 |
| **RA/Collagen Vascular Diseases**  **Total**  **Active** | 45  15 | 0.20  0.20 | 1.00  0.99 | 1.00  0.33 | 0.94  0.98 |
| **Renal failure**  **Total**  **Active** | 8  3 | 0.13  0.33 | 1.00  1.00 | 0.50  0.50 | 0.99  1.00 |
| **Solid Tumor w/o Metastasis**  **Total**  **Active** | 46  23 | 0.39  0.74 | 0.99  0.99 | 0.78  0.74 | 0.95  0.99 |
| **Valvular Disease**  **Total**  **Active** | 5  3 | 0.60  0.67 | 1.00  0.99 | 0.60  0.40 | 1.00  1.00 |
| **Weight Loss**  **Total**  **Active** | 222  193 | 0.02  0.02 | 0.98  0.98 | 0.40  0.30 | 0.61  0.66 |

^α^ Denotes total number of patients with the specified comorbidity. 429 patients presented with at least 1 Elixhauser comorbidity. However, sum of patients with each comorbidity type does not equal 429 as patients may have > 1 Elixhauser comorbidity.

^β^ Comorbidities stratified in chart review by activity on admission. Active comorbidities are those requiring specific management at the time of admission.

^*^ No patients identified with comorbid AIDS, lymphoma, psychoses, or paralysis.

**References**

1. Dindo D, Demartines N, Clavien PA: Classification of surgical complications: a new proposal with evaluation in a cohort of 6336 patients and results of a survey. *Ann Surg* 2004, 240(2):205-213.
